# Suspected Cauda Equina Syndrome: Can the Presence of an Orthopaedic Doctor in the Emergency Department Reduce Waiting Times for MRI Scans and Inpatient Stays?

**DOI:** 10.7759/cureus.49284

**Published:** 2023-11-23

**Authors:** Abdelwakeel Bakhiet, Hatem Elsayed, Khadija Elamin, Upamanyu Nath, Mohamad Alqubaisi, Abhirun Das, Anand Pillai

**Affiliations:** 1 Trauma and Orthopaedics, Wythenshawe Hospital, Manchester University NHS Foundation Trust, Manchester, GBR

**Keywords:** spinal services, mri scan, orthopaedic emergency, decrease waiting times, cauda equina syndrome

## Abstract

Introduction

Cauda equina syndrome (CES) is a very rare but devastating surgical emergency that can lead to permanent bowel, bladder or sexual dysfunction and lower limb paralysis. Although it is a clinical syndrome, an MRI scan is a critical diagnostic investigation for these patients and should be done as soon as possible. Our hospital is a district general hospital with no spinal services on site. There is a protocol in place for the management of these patients locally with MRI scanning during daytime hours. However, if the patient presents after 8 pm, they are discussed with the tertiary spinal centre, which then advises if the patient requires transfer overnight for urgent scanning. Considering an MRI scan is a critical diagnostic step for these patients, we introduced a role for an orthopaedic doctor in the Emergency Department (ED) to assess all of these patients before collecting data for the second cycle. The aim of this audit was to see the effect of having an orthopaedic doctor in the ED for the assessment of these patients and its impact on waiting times and admission rates.

Methods

A closed-loop audit cycle was done looking into all referrals to trauma and orthopaedics with acute back pain and suspected CES in a district general hospital with no spinal services on site. The first cycle was between September 30, 2020, and May 31, 2021, and included 93 patients in total. Following this, a role for an orthopaedic doctor in ED was introduced from December 1, 2021, to January 31, 2022, for assessment of these patients. Data was then collected retrospectively for all patients referred during this period (n=36). Data was extracted from all relevant clinical systems including electronic patient record (EPR), Patient Pass (Patient Pass Ltd, Greater Manchester, England), which is the system used to digitally communicate with regional spinal services, and PACS (picture archiving and communication systems). The data was collated on a Microsoft Excel spreadsheet (Microsoft Corporation, Redmond, Washinton, United States) and analysed.

Results

Data were collected for a total of 36 patients in the second cycle following the introduction of an orthopaedic doctor in the ED. The age of patients referred was 30-89 years with a mean age of 51; 44.4% were male (n=16) and 55.5% female (n=20). All the patients who were referred received their MRI scan and report within 24 hours of presentation to the ED. In the first cycle of the audit, the mean waiting time for an MRI scan had been 12.5 hours, which was reduced to eight hours following the introduction of an orthopaedic doctor in the ED during daytime hours. This was stratified further according to the time patients presented to the hospital. From 8 am to 4 pm, the mean waiting time for an MRI scan was 9.5 hours pre-intervention and 5.5 hours after. From 4 pm to 12 am, the mean waiting time was 18 hours before and 13 hours after, and from 12 am to 8 am, the waiting time for scans improved from 8.5 hours to 6.5 hours. The number of patients discharged on the same day greatly improved from 29% (n=27) in the initial study to 58% (n=21). This decreased unnecessary inpatient stays from 71% (n=66) to 42% (n=15).

Conclusion

This study showed that an orthopaedic doctor in the ED for the acute assessment of patients referred with possible CES is an effective way of improving their management. This decreases waiting times for MRI scans and therefore allows the patient to be managed more efficiently.

## Introduction

The leading cause of disability in the United Kingdom (UK) as per the National Institute of Clinical Excellence (NICE) and the UK Spine Societies Board is back pain, accounting for 11% of the total UK disability population [1,2]. However, despite the implementation of a national back pain pathway, referrals to spinal surgery are increasing every year whilst waiting times for assessment and treatment are longer than 18 weeks [3].

Cauda equina syndrome (CES) is a very rare (one to three in 100,000 population) but devastating surgical emergency that is caused by compression of the terminal nerve roots in the lower lumbar spinal canal [1]. This presents clinically with a constellation of symptoms including back pain ± bilateral leg weakness ± bilateral leg paraesthesia ± bowel dysfunction ± bladder dysfunction ± saddle anaesthesia ± sexual dysfunction [3]. Missed or delayed diagnosis can lead to catastrophic consequences such as permanent loss of bowel or bladder function, loss of sexual function, and lower limb paralysis [4,5]. 

CES can be subdivided due to the degree of neurological deficit. Those affected with only bilateral radiculopathy and/or subjective urinary/bowel dysfunction or perineal anaesthesia and no objective evidence of CES would be classified as CES-Suspected (CES-S). Those with objective symptoms of CES but have normal bowel or bladder function are classified as CES-Incomplete (CES-I), and those with objective symptoms and with urinary retention and overflow incontinence are classified as CES-Retention (CES-R) [6]. Those with CES-I at the time of surgery have a more favourable diagnosis than those with CES-R at the time of decompression as they are less likely to have irreversible damage to nerve roots [7]. It has been described as a “tragedy” that a patient should progress from CES-I to CES-R whilst under medical supervision and is usually preventable [8].

Despite the list of “red flag symptoms” for CES, clinical assessment is only reliable when symptoms and signs are often irreversible [9]. Therefore, although it is a clinical syndrome, an MRI scan is a critical diagnostic investigation for these patients and should be undertaken as soon as possible, with initiatives such as the National Suspected CES Pathway recommending that all MRI scans for suspected CES are done locally within four hours of request to radiology by June 2024 [5]. Where this is not possible currently, there should be protocols for discussion with local spinal and radiology services to facilitate urgent out-of-hours scanning [5].

Once proven on an MRI scan, CES requires surgical decompression to relieve the pressure on the terminal roots of the spinal cord, although the urgency still remains a topic of debate depending on whether it is CES-S, CES-I, or CES-R [9]. However, the advice given by the British Association of Spinal Surgeons (BASS) [10], NICE [2], and the Society of British Neurological Surgeons (SBNS) [11] are to operate on CES patients as soon as practically possible to prevent any further neurological deterioration and to maximise recovery potential.

CES, although rare has a disproportionately high rate of medico-legal claims with an average payment of £117,331 per case [12]. Therefore it is all the more important to try and reduce the risk of permanent disability to these patients by ensuring timely referral to appropriate services, diagnosis by MRI scan and surgical decompression [13].

Our hospital is a district general hospital with no spinal services on site but acutely manages patients presenting with suspected CES. Therefore we have a protocol in place for the management of these patients. The patient presents to the emergency department (ED) on their own accord, or by referral to the trauma and orthopaedics on-call team either by their general practitioner (GP) or another district general hospital that does not have MRI scanning capabilities. They are assessed in the ED and during daytime hours an MRI scan is requested prior to discussion with the regional tertiary spinal centre. However, if the patient presents after 8 pm, they are discussed with the tertiary centre immediately from the ED, which then advises whether the patient needs an urgent overnight MRI scan, in which case they are transferred, or if they are to remain locally for scanning during daylight hours. If CES is excluded on the MRI scan, then the patient is either discharged with appropriate follow-up or is admitted to the ward for pain management and therapy until they are safe for discharge. The lack of 24-hour access to MRI scanning facilities in our hospital for these patients makes it all the more important that they are accurately assessed in a timely manner in the ED so that appropriate investigations can be requested and spinal services contacted.

We conducted a clinical audit initially looking into the efficiency of these patients’ assessment and their waiting times for MRI scans. We then introduced an intervention before re-auditing to close the loop. This article discusses the impact of our interventions and presents the results of this closed-loop audit.

## Materials and methods

A closed-loop audit cycle was completed looking into all referrals to trauma and orthopaedics with acute back pain and suspected CES in a district general hospital, Wythenshawe Hospital, Manchester, UK, with no spinal services on site. This study received approval from the local clinical audit department at Wythenshawe Hospital and the audit approval number was S024. Ethical approval was not required as there was no direct involvement of human subjects, and data was collected for quality improvement and audit purposes.

A conclusive list of patients was provided by the orthopaedic trauma co-ordinators who keep a comprehensive record of all the patients that were referred to the trauma and orthopaedics team for both cycles of this project. Data was extracted from all relevant clinical systems including the electronic patient record (EPR), Patient Pass (Patient Pass Ltd, Greater Manchester, England), the system used to digitally communicate with regional spinal services, and medical imaging software PACS (picture archiving and communication systems). The data was collated on a Microsoft Excel spreadsheet (Microsoft Corporation, Redmond, Washington, United States) and analysed.

The first cycle was between September 30, 2020, and May 31, 2021, and included 93 patients in total. Data was collected retrospectively for these patients and analysed accordingly. Following this we hypothesized that the presence of a middle-grade trauma and orthopaedic specialist doctor in the ED as part of the urgent treatment centre would facilitate faster and more accurate assessment of these patients, improve waiting time for MRI scans, and ensure that these facilities are used appropriately. This role was introduced from December 1, 2021, to January 31, 2022, and following this we conducted our second cycle. Data was collected retrospectively after the second cycle for 36 patients comprising all patients referred to the on-call orthopaedic team during this period. We audited patient demographics, symptoms and clinical signs on presentation, length of stay, waiting time for MRI scan, the outcome of the scan, and the outcome following referral to spinal specialist services. All patients with acute back pain and suspected CES were included and those with fractures were excluded. We used NICE, BASS, and SBNS guidelines, and locally agreed trust protocols as a standard of reference for this study [2,10,11].

## Results

Data was collected for a total of 36 patients in our second cycle following the introduction of an orthopaedic doctor in the ED for the assessment of these patients. The age of patients referred was between 30 and 89 years with a mean age of 51. Considering gender, the distribution was 44.4% male (n=16) and 55.5% female (n=20).

**Figure 1:**
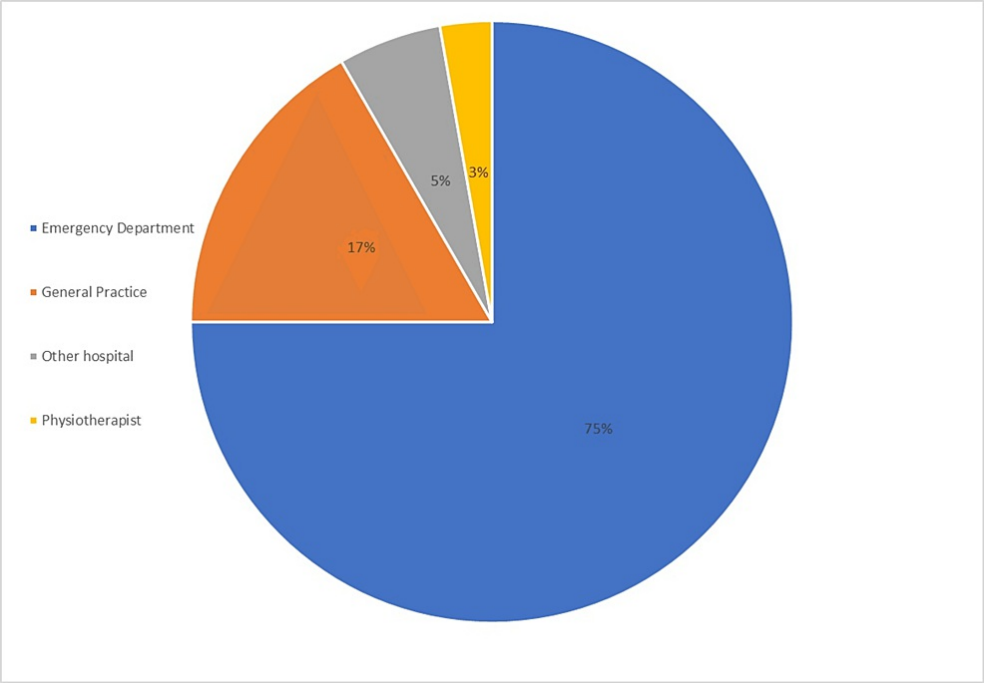
Distribution of different sources of referrals for patients with suspected CES CES: cauda equina syndrome

The majority of the referrals were received from the ED, with 75% of referrals (n=27). The remainder of the referrals received were from general practices with 16.7% (n=6), 5.5% of referrals were from another district general hospital with no MRI scanning facilities (n=2), and 2.7% of referrals were from local physiotherapists (n=1) (Figure 1). Most patients presented during daytime hours with 58.3% of patients presenting to the hospital between 8 am and 4 pm (n=21); 33.3% presented from 4 pm to 12 am (n=12) and 8.3% presented between 12 am and 8 am (n=3). 

Of the total patients, 61% presented with a bilateral lower limb sensory deficit (n=22) while 56% presented with a motor deficit (n=20). Digital rectal examination (DRE) examination was abnormal in 32% of patients (n=11, two patients declined DRE), with 47% of patients complaining of bladder dysfunction (n=17) and 14% with bowel dysfunction (n=5) (Table 1).

**Table 1:** Presenting symptoms of the patients with suspected CES CES: cauda equina syndrome

Symptoms/signs	No. of patients	Percentage
Sensory deficit	22 patients	61%
Motor deficit	20 patients	56%
Digital rectal examination abnormal	11 patients (2 declined)	32 %
Bladder dysfunction	17 patients	47%
Faecal incontinence	5 patients	14%

All of the patients who were referred received their MRI scan and report within 24 hours of presentation to the ED. In the first cycle of the audit, the mean waiting time for an MRI scan was 12.5 hours and this reduced to eight hours following the introduction of an orthopaedic doctor in the ED during daytime hours. We stratified this further according to the time that patients presented to the hospital and found that from 8 am to 4 pm, the mean waiting time for an MRI scan was 9.5 hours pre-intervention and 5.5 hours after. From 4 pm to 12 am, it was 18 hours before and 13 hours after, and from 12 am to 8 am, the waiting time for scans improved from 8.5 hours to 6.5 hours. The number of patients who were discharged on the same day greatly improved from 29% in our initial study (n=27) to 58% (n=21). This reduced the need for unnecessary inpatient stays from 71% (n=66) to 42% (n=15) (Table 2).

**Table 2:** Waiting times and rates of discharge or admission for patients with suspected CES in the pre-intervention and post-intervention groups CES: cauda equina syndrome

	1^st^ Cycle	2^nd^ Cycle
Mean waiting time	12.5 hours	8 hours
Mean waiting time 8am-4pm	9.5 hours	5.5 hours
Mean waiting time 4pm-12am	18 hours	13 hours
Mean waiting time 12am–8am	8.5 hours	6.5 hours
Patients discharged same day, n (%)	27 (29%)	21 (58%)
Patients requiring a hospital bed	71%; average 11 patients/month	42%; average 7 patients/month

We followed up with the patients in our second cycle to see the outcomes of their MRI scans. Two of the patients were transferred to the regional spinal centre for overnight assessment and MRI scanning: one was repatriated to our hospital after CES was excluded, and the other was admitted for further assessment. No patients were found to have CES. Possible CES was identified in 3% of patients (n=1), and no evidence of CES was found in 97% of cases (n=35).

## Discussion

The purpose of this study was to assess how long patients who were referred for suspected CES had to wait for their MRI scan and to study the impact of having an orthopaedic doctor present in the ED for the assessment of these patients on this. It is important to ensure that MRI scanning resources are used appropriately to ensure that these patients receive their scans as soon as possible, and even within the stated four-hour target from when it is requested [1,5].

In our first cycle, we noticed that the waiting times for MRI scans were variable depending on the time the patient was initially seen in the ED and referred to orthopaedics. Whilst most tertiary centres are able to perform MRI scans 24 hours a day, most district general hospitals do not have access to MRI scanning from 8 pm onwards and thus delays in referrals to orthopaedics from the daytime meant that patients would have to wait until the morning for their scan [14]. This requires the patient to be admitted overnight for observation and therefore means more hospital beds are utilised, possibly unnecessarily.

On receiving these patients in the hospital, every effort should be made to expedite their management, to avoid CES-I from becoming CES-R under the supervision of medical professionals as the former has a much more favourable prognosis [7]. Furthermore, studies have found that all of the symptoms and signs of CES have a poor predictive value and thus an MRI is critically important for diagnosis [14]. The literature shows that delayed intervention can lead to irreversible neurological damage; whilst the timing of surgery is still controversial, most studies recommend that this is achieved within 48 hours [1,5,12]. This stimulated us to analyse the waiting times for MRI scans for these patients in our department and start an intervention to improve these as they are a critical part of their management.

Following the introduction of an orthopaedic doctor in the ED, the waiting times for MRI scans for these patients greatly improved with 29% more patients being discharged the same day. We believe this was due to accurate and prompt assessment, which permitted earlier MRI requests as a direct result; faster diagnosis and management. This is similar to findings in Nasim et al.’s paper where it was shown that the earlier the MRI scans are requested upon initial patient assessment, the shorter the inpatient stay for these patients [15].

This study was a closed-loop audit which included all patients that were referred with possible CES without fractures to the orthopaedic service. It highlighted the effect of accurate and timely specialist assessment in triaging these patients appropriately to ensure they receive their MRI scans as quickly as possible. This has been shown to have a direct effect on reducing waiting times and inpatient stays, ultimately meaning fewer hospital beds are used unnecessarily.

However, considering that the study was retrospective, the accuracy of previous documentation directly affected the accuracy of data collection. Therefore, there is a possibility of inaccurate information or misinterpreted clinical findings by medical professionals. Considering that those who collected and analysed the data had no control over the accuracy of documentation, this poses a further limitation. Lastly, the relatively small sample size may hinder the accuracy of the results.

## Conclusions

We believe that having an orthopaedic doctor in the ED for the acute assessment of patients referred with possible CES is an effective way of improving their management. We have shown that this decreases waiting times for MRI scans and therefore allows the patient to be managed more efficiently.

Furthermore, it reduces the amount of unnecessary admissions and the number of beds occupied by these patients. Due to concerns about the steady rise in the number of patients requiring orthopaedic assessment, we should try to find more efficient and effective techniques or pathways to deliver optimal care for these patients.
